# Linkage of alternative exon assembly in *Drosophila TrpA1* transcripts

**DOI:** 10.1016/j.mocell.2024.100110

**Published:** 2024-09-11

**Authors:** Eun Jo Du, MinHyuk Lee, Seon Yeong Kim, Se Hoon Park, Hye-Jung Ohk, KyeongJin Kang

**Affiliations:** 1 Neurovascular Unit Research Group, Korea Brain Research Institute, Daegu 41062, Republic of Korea; 2 Department of Brain Sciences, DGIST, Daegu 42988, Republic of Korea

**Keywords:** Alternative RNA splicing, Chemical nociception, Mutually exclusive exons, Splicing coordination, Transient receptor potential ankyrin 1

## Abstract

*Drosophila TrpA1* (*transient receptor potential ankyrin 1*) transcripts are alternatively spliced at 2 distinct sites each with a choice of mutually exclusive exons. The first site determines exon1 encoding the amino terminus to produce either nucleophile-, electrophile- and noxious temperature-gated TRPA1(A) or electrophile- and innocuous warmth-gated TRPA1(B). The second site selects for exon10, resulting in *TrpA1* variants with either exon10a or exon10b encoding a domain between the N-terminal ankyrin repeats and the transmembrane segments. Although unbiased assembly would generate TRPA1 with 4 different domain combinations, the functional impact of these alternative domains remains to be thoroughly examined. Here, we find that there is a relatively strong linkage in mRNA splicing between the 2 sites in the case of *TrpA1(B)*, but not *TrpA1(A)*, transcripts. Our semiquantitative assay, consisting of reverse transcription polymerase chain reaction and Sanger sequencing, revealed that exon10b is little coupled with *TrpA1(B)* transcripts, suggesting that only 3 isoforms, TRPA1(A)-exon10a [denoted as TRPA1(A)], TRPA1(A)-exon10b [TRPA1(A)10b], and TRPA1(B)-exon10a [TRPA1(B)], are present at detectable levels using our method. Interestingly, heterologously expressed TRPA1(A)10b showed elevated sensitivity to low concentrations of N-methyl maleimide, a cysteine-modifying electrophile, compared with other isoforms. Equivalent isoforms in malaria-transmitting *Anopheles gambiae* displayed a similar pattern of isoform-dependent N-methyl maleimide dose dependences, suggesting that the chemosensory regulation by selective domain assembly is conserved in insect TRPA1s. Thus, alternative RNA splicing of exon10 is coordinated in conjunction with the first exons, regulating chemical sensitivity of insect TRPA1s.

## INTRODUCTION

A few members of the transient receptor potential (TRP) ion channel family have been identified as sensory receptors and transducers for various environmental stimuli (Julius, 2013, Patapoutian et al., 2003) since the isolation of the naturally occurring *Drosophila melanogaster trp* mutant, which is characterized by transient photoreceptor potential responses (Cosens and Manning, 1969). Among sensory TRP channels, transient receptor potential ankyrin 1 (TRPA1) is unique in that the channel protein is activated in response to reactive electrophiles (Hinman et al., 2006, Macpherson et al., 2007), which typically inactivate key biomacromolecules and may inflict tissue damage in organisms. TRPA1 has been shown to be conserved across Bilateria as a receptor for tissue-damaging chemicals, sharing a mechanism by which TRPA1 directly detects reactive chemicals that covalently modify intracellular cysteines (Kang et al., 2010). However, the thermal sensitivity of TRPA1 diverges among animals (Gracheva et al., 2010, Hamada et al., 2008, Panzano et al., 2010, Viswanath et al., 2003, Wang et al., 2009). TRPA1s from snakes and insects are activated by warm temperatures, while those from mammals are responsible for the perception of noxious cold temperatures.

Isoform diversity of TRPA1 has been recently reported in mice (Zhou et al., 2013) and fruit flies (Kang et al., 2012; Kwon et al., 2010; Zhong et al., 2012). In mice, functional expression of a newly identified splicing variant depends on its physical association with the classical TRPA1 isoform. These heterotetrameric complexes showed elevated sensitivity to reactive electrophiles in sensory neurons. Sensory discrimination between chemical and thermal cues that commonly activate a single sensory TRP channel is important in particular when the TRP channel is a receptor shared for both noxious and innocuous sensory inputs, as behavioral responses to the 2 stimuli must be distinct for animals’ well-being. *Drosophila melanogaster* achieves sensory discrimination between noxious chemicals and innocuous warm temperature, both of which activate TRPA1, by expressing 2 different isoforms in distinct sensory circuits. The 2 isoforms arise from the alternative use of the first exons with different 5′ UTRs (untranslated regions) and start codons, producing TRPA1(A) and TRPA1(B) with distinct N-termini (Kang et al., 2012) (Supplemental Fig. 1). When tested in *Xenopus* oocytes, the thermosensitivity of TRPA1(A) was much suppressed compared with TRPA1(B), while both isoforms respond to electrophilic chemicals with similar sensitivities. Recently, TRPA1(A) was reported to be thermosensitive as well with a thermal activation threshold at ∼42℃, mediating nocifensive behavior in larvae (Gu et al., 2019, Gu et al., 2022). However, at innocuous temperatures, TRPA1(A) is activated in response to the nucleophilicity of reactive chemical compounds (Du et al., 2016a, Du et al., 2016). This characteristic makes TRPA1(A) an excellent receptor of reactive oxygen species (ROS) and free radical-generating electromagnetic illumination, such as ultraviolet light, compared to electrophile-only sensitive TRPA1(B), as ROS and radicals often possess nucleophilicity as well as electrophilicity. Interestingly, it was found that pigments generating ROS upon light absorption also activate TRPA1(A) depending on the nucleophile responsiveness of the ion channel (Du et al., 2019). This capability did not require light illumination, suggesting that TRPA1(A) detects chemical compounds with potential phototoxicity through nucleophile sensitivity as well as the photochemical products such as radicals and ROS as described earlier.

In addition, a small region encoded by mutually exclusive exons (referred to as exon10a and exon10b in this study) was proposed to determine heat sensitivity of TRPA1 (Supplemental Fig. 1) (Zhong et al., 2012). We refer to this domain “BAT,” as it is localized *b*etween N-terminal *a*nkyrin repeats and the *t*ransmembrane segments of *Drosophila* TRPA1. Here, we analyzed the representation of alternative exons at the 2 loci of *TrpA1* transcripts by a simple molecular biological approach and found that exon10b is little associated with thermo-sensitive TRPA1(B) and modulates chemical sensitivity of TRPA1(A). Our results indicate that alternative splicing of exon10 in the *TrpA1* transcript is coordinated to implement exon10b for chemosensory function of TRPA1.

## RESULTS AND DISCUSSION

**Fig. 1:**
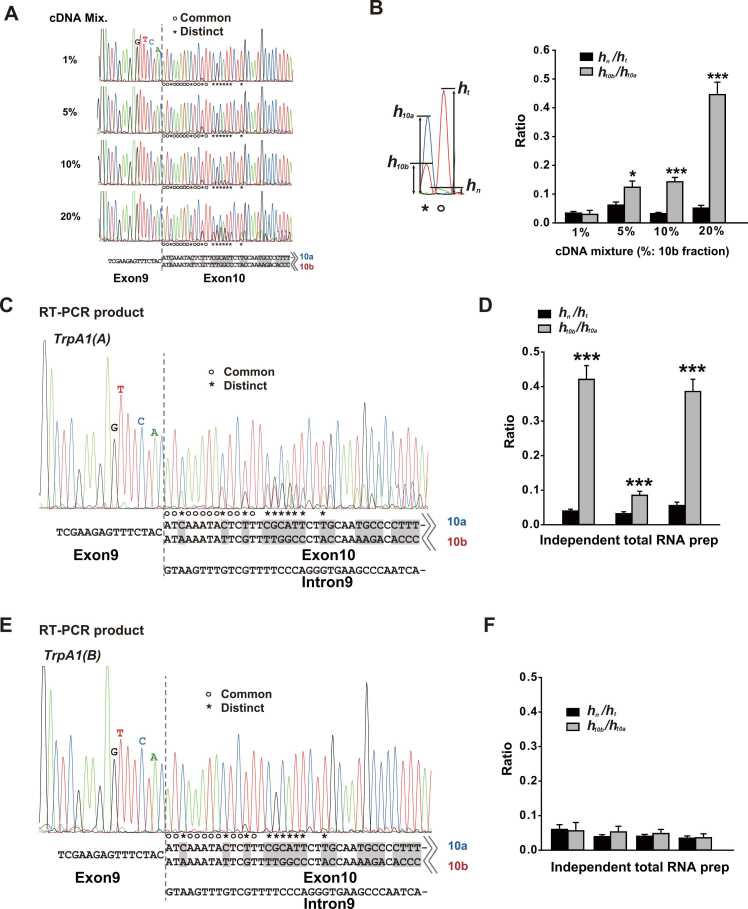
The transcript variants of *TrpA1(A)* not *TrpA1(B)* can contain either exon10a or exon10b. (A and B) Occurrence of combined exon10a and exon10b cDNAs can be semiquantitatively assessed by conventional Sanger nucleotide sequencing. (A) Representative sequencing results. The dashed vertical line marks the exon junction of exon9 and exon10. Bases common and distinct between the alternative exons were indicated by “○” and “*,” respectively. For sequencing reactions, nested-F oligomers (Supplemental Fig. 1) were used. (B) Ratios calculated for estimation of exon10b frequencies are monotonically proportional to the exon10b fraction in the mixture. The ratios of h_n_/h_t_ and h_10b_/h_10a_ indicate the reaction noise for common bases and exon10b frequency for distinct bases, respectively. *P < .05, ***P < .001, Student’s t-test. (C and E) Representative sequencing results of PCR fragments primed with either 1a-F (*TrpA1(A)*) or 1b-F (*TrpA1(B)*). For sequencing reactions, nested-F oligomers were used. (D and F) Each experiment with independent total RNA preparation was shown. ***P <.001, Student’s t-test. RT-PCR, reverse transcription polymerase chain reaction. cDNA, complementary DNA.

Before nucleotide sequencing-based quantitation of alternative exons included in *TrpA1* transcripts, we assessed how quantitative the conventional nucleotide sequencing results are (Fig. 1A and B). Mixtures of the plasmids included 1% to 20% of the cloned full-length *TrpA1* cDNA (complementary DNA) with exon10b as fractions of that with exon10a and were subjected to nucleotide sequence determination by the Sanger sequencing method. In the sequencing results presented in Figure 1A, bases common between two exons are indicated by circles (○), and those that differ are marked by asterisks (*). The first 10 common and distinct bases were analyzed (in total 20 bases), because farther downstream base peaks of exon10a and exon10b sequencing products are not well aligned for relative quantitation due to growing differences in the molecular weights of electrophoretically migrating exon10a and exon10b sequencing fragments. To assess the quality of sequencing reactions, background reaction levels were estimated by calculating the ratio of the highest noise peak (h_n_) to the peak of the nucleotide base read (h_t_) for the first 10 common bases (Fig. 1B). For the estimation of exon10b representation in the sequencing results, the fluorescence intensities of the exon10b sequence (h_10b_) were divided by those corresponding to exon10a (h_10a_). Except for the mixture containing 1% of exon10b cDNA, the 3 other conditions showed statistically significant and somewhat proportionally increasing differences between the ratios resulting from background reaction noise and the presence of exon10b (Fig. 1B, right). Thus, fractions of exon10b cDNA equal to or greater than 5% in the total population of *TrpA1* cDNA can be detected through nucleotide sequencing.

To appraise the frequency of exon10a and exon10b spliced with either exon1a or exon1b, cDNAs polymerase chain reaction (PCR)-amplified with transcript variant-specific primers were subjected to Sanger sequencing. The relative splicing frequencies of exon10a and exon10b were determined by comparing the heights of peaks corresponding to exon10a or exon10b sequences as described earlier. The straddling primers effectively suppressed PCR amplification of genomic sequences, as fluorescence peaks corresponding to the intron9 sequence were hardly detectable (Fig. 1C and E). Reactions with the *TrpA1(A)*-specific 1a-F primer yielded reverse transcription polymerase chain reaction (RT-PCR) products containing both exon10a and exon10b sequences in experiments with 3 independent total RNA preparations (Fig. 1C and D), indicating that *TrpA1(A)* transcripts with exon1a can be spliced with either exon10a or exon10b. Although the experiment with a cDNA preparation displayed in the middle of Figure 1D showed a low frequency of exon10b-specific bases compared with the other 2 experiments, exon10b-specific bases were still discerned with strong statistical significance when compared with sequencing peaks derived from background noise. In contrast, PCR products primed with the *TrpA1(B)-*specific 1b-F oligonucleotide did not show peaks of exon10b-specific bases significantly higher than the background level in 4 experiments with independent total RNA preparations (Fig. 1E and F). These results indicate that alternative exons at 2 different loci in the *Drosophila TrpA1* transcript are not unbiasedly combined to produce all 4 possible combinations and that exon10b is little spliced in with exon1b of *TrpA1(B)* that encodes the highly thermosensitive warmth-gated TRPA1(B) isoform (Kang et al., 2012). Although *TrpA1(B)-*exon10b can be present in less than 5% of the whole population of *TrpA1(B)* transcripts, the low occurrence of this combination suggests that the control of TRPA1(B) thermosensitivity by exon10b might be restricted to few cells in flies. *TrpA1(B)* is a primary transcript detected in the head that lacks the proboscis (Kang et al., 2012). Fisher’s exact test regarding successful detection of exon10b produced *P* < .05 between the experiments of Figure 1D and F, further supporting the difference of exon10 splicing between *TrpA1(A)* and *TrpA1(B)* variants. The fact that only a few cells in the brain have been described as expressing TRPA1 related to warmth sensitivity (Hamada et al., 2008) suggests that the scarcity of *TrpA1(B)10b* led to the failure of its detection in our experiments although it may encode an innately expressed ion channel. However, its rarity relative to total *TrpA1(B)* transcripts, which were readily RT-PCR-amplified in our assay, also raises questions about its physiological significance. It is possible that preferential PCR amplification for exon10a in the *TrpA1(B)* cDNA might have biased the estimation of the exon10b frequency in *TrpA1(B)* cDNA. Differences in GC contents and amplicon sizes can result in uneven amplification between the 2 transcript variants (Walsh et al., 1992). However, the GC contents and lengths of the 2 amplicons are very similar: 50% and 51% of GC pairs and 468 and 465 bases long for exon10a and exon10b amplicons, respectively. Note that the PCR primers (Nested-F and Nested-R) for final reactions are common for both exon10s, as they anneal in exons shared in all transcripts (Supplemental Fig. 1A and C).

**Fig. 2:**
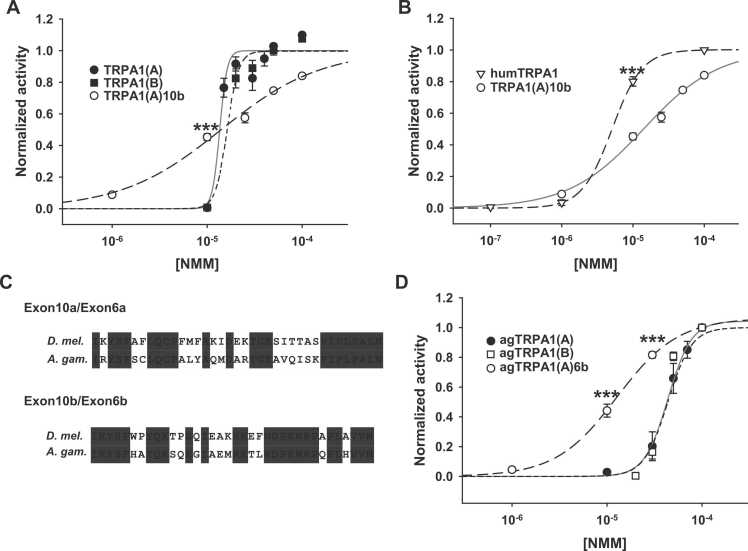
Exon10b or exon6b-encoded BAT modifies the responsiveness of fruit fly and mosquito TRPA1(A)s to N-methyl maleimide compared with other isoforms containing the exon10a or exon6a-encoded domain. (A) Averaged dose dependence data of TRPA1 isoforms. (B) Comparison of averaged dose dependences of humTRPA1 and TRPA1(A)10b. (C) Sequence alignment of exon10- and exon6-encoded domains. (D) Averaged dose dependence data of agTRPA1 isoforms. ***P < .001, ANOVA Tukey or Student’s t-test, depending on the number of compared groups. ANOVA, analysis of variance.

In that exon10b could be included as part of *TrpA1(A)* previously shown to produce a receptor for reactive chemical compounds, BAT encoded by exon10b might play a primary role in modulating the reactive electrophile detection of TRPA1(A). To examine the functional effect of exon10b-encoded BAT in the TRPA1(A) isoform (TRPA1(A)10b) in comparison with other TRPA1 isoforms, each *in vitro-*transcribed capped RNA of 3 transcript variants was injected into *Xenopus laevis* oocytes for electrophysiological characterization in response to the reactive electrophile N-methyl maleimide (NMM). NMM is a TRPA1 agonist well-known for its electrophilic reactivity toward sulfhydryl groups of cysteines. By covalently modifying a specific set of cysteines in the N-terminal domain, NMM and other electrophilic TRPA1 agonists, such as allyl isothiocyanate, activate the ion channel (Hinman et al., 2006, Macpherson et al., 2007). Note that TRPA1 isoforms with BAT encoded by exon10a are referred to as TRPA1(A) or TRPA1(B) for simplicity. In contrast to previous studies (Kang et al., 2010, Kang et al., 2012), in which dose dependences of TRPA1 isoforms were obtained with NMM application for a fixed time length, NMM perfusion was switched to the next higher concentration after the current was close to saturation at the currently given NMM concentration, to reduce the experimental variation caused by differences in the perfusion flux rate. Responses from cells expressing either TRPA1(A), TRPA1(B), or TRPA1(A)10b were close to the maximum at 0.1 mM NMM (Supplemental Fig. 2A-C). TRPA1 isoforms with exon10a BAT showed large steps of current increases at NMM concentrations 15 or 20 μM (Fig. 2A). The further increase of NMM concentrations did not induce dramatic increases of the TRPA1 current (Fig. 2A, Supplemental Fig. 2A and B). This might be because the efficacy of NMM is indifferent, after intracellular sulfhydryl groups, which are more reactive than those of TRPA1, are already occupied by the irreversible cysteine modifier NMM. As previously reported (Kang et al., 2012), the sensitivities of TRPA1(A) and TRPA(B) acquired with our current-saturating approach were similar to each other (Fig. 2A, EC50s: 13.6 ± 0.6 and 16.3 ± 1.2 μM, respectively). Interestingly, cells expressing TRPA1(A)10b exhibited current increases at 1 and 10 μM NMM (Fig. 2A, Supplemental Fig. 2C) with a wide dynamic range spanning between 1 and 100 μM compared to other variant channels, although EC50s were similar among all isoforms (EC50 of TRPA1(A)10b: 14.1 ± 2.7 μM). Comparison of fruit fly TRPA1 with human TRPA1 (humTRPA1) reveals that the NMM sensitivity of TRPA1(A)10b is closer to that of humTRPA1 at low NMM concentrations than other isoforms (Fig. 2B, Supplemental Fig. 2C and D; EC50s of humTRPA1: 5.1 ± 0.5 μM). Thus, the exon10b-encoded domain, alternatively assembled for the TRPA1(A) isoform, is implemented to substantially modify the profile of TRPA1(A) responses to the reactive chemical NMM. We cannot exclude the possibility that TRPA1(B)10b exists in the fly in limited circumstances as discussed earlier. Although it is expected that coupling the exon10b BAT with TRPA1(B) will result in a similar shift in electrophile sensitivity based on our results, the presence of *TrpA1(B)* in the chemosensory cells has not been established to date (Kang et al., 2012). Thus, if TRPA1(B)10b indeed occurs in flies, exon10b BAT might rather serve as a regulatory domain for the temperature-sensing ability of TRPA1(B) as proposed in a previous study (Zhong et al., 2012). Modulation of TRPA1 chemosensory function by alternative assembly of the exon10-encoded domain appears to be conserved across insect species, as deduced insect TRPA1 sequences were well aligned with amino acid sequences of *Drosophila* BAT (Supplemental Fig. 3, flybase.org/blast/). To test if exon10b-equivalent BATs from other species affect TRPA1 in a similar manner, TRPA1 isoforms from the malaria-transmitting mosquito *Anopheles gambiae* were examined for NMM sensitivities. Although the *A. gambiae TrpA1* (*agTrpA1)* gene contained fewer exons than *D. melanogaster TrpA1,* the alternative exons encoding the N-terminus (Kang et al., 2012) and BAT are organized as in *D. melanogaster TrpA1* (Supplemental Fig. 1C and D) and the primary sequences of mosquito and fly BATs showed high similarities (Fig. 2C). Overall dose dependences of the isoforms with exon6a BAT are inseparable (Fig. 2D, Supplemental Fig. 2E and F; EC50s of agTRPA1(A) and agTRPA1(B): 43.8 ± 3.9 and 39.5 ± 1.1 μM, respectively), although agTRPA1(A) exhibited slightly higher NMM sensitivity at concentrations of 10 and 30 μM NMM than agTRPA1(B) in some cells (Supplemental Fig. 2E and F). The effect of alternative BATs is more pronounced for agTRPA1(A) compared to *Drosophila* TRPA1(A). The agTRPA1(A)6b isoform showed higher relative sensitivities at most NMM concentrations (EC50: 12.7 ± 1.2 μM) with the NMM dose dependence left-shifted, compared to exon6a isoforms (Fig. 2D). The dynamic range of the channel response to NMM is also wider than the 6a BAT isoforms but narrower than fruit fly TRPA1(A)10b (Fig. 2D). These results indicate that the alternative BAT assembly in fruit fly and mosquito TRPA1s modifies the response of the channel to the cysteine-modifying electrophile NMM, demonstrating the conserved role of the alternative RNA splicing for the chemosensory TRPA1 function in insects. As it is well established that TRPA1 is activated by the electrophilic reactivity of NMM (Hinman et al., 2006), similar sensitivity differences for other electrophilic reactive chemicals, such as allyl isothiocyanate, are expected between TRPA1 isoforms with either exon10a or exon10b BAT. Taken together, the mutually exclusive exons encoding BAT are alternatively spliced for *TrpA1(A)*, not *TrpA1(B)*, and modulate chemosensory function of TRPA1(A) in insects.

Many genes appear to be under the control of multiple alternative splicing events (Hao et al., 2023; Kwon et al., 2023; Lee et al., 2024), but how alternative splicing is coordinated in relation to other sites of alternative exons, and what the functional outcome of such coordination is, has yet to be unraveled. The *Caenorhabditis elegans* BK (big potassium) channel gene *slo-1* provides another example of multiple splicing coordination (Glauser et al., 2011), where the frequencies of exons in the transcripts were correlated with the combinatory coappearance of separate alternate exons. A mutation that disrupts a UAAAUC element enriched in the flanking introns impaired the coordination, and the UAAAUC element was proposed to be the putative *cis*-regulatory element critical for the splicing coordination. In the *Drosophila TrpA1* gene, however, the element was not found in adjacent introns, suggesting that the splicing coordination observed for *TrpA1* is accomplished by a molecular mechanism distinct from that involving the UAAAUC element. Further studies, which our results would provide a start point for, are required to identify the essential splicing factors and elements that regulate the splicing coordination of *TrpA1*. Given the evolutionary conservation of the chemosensory role and the divergence of thermosensory role of *TrpA1*, the chemosensory function of TRPA1(A) likely precedes the thermal sensitivity of TRPA1(B) in the course of evolution. Therefore, it is conceivable that the *TrpA1(B)-*specific sequence that has recently been added to the *TrpA1* gene might carry elements that suppress the inclusion of exon10b to facilitate the thermosensory function of TRPA1. Thus, the *TrpA1(B)-*specific genome sequence would be a primary target for an initial survey for splicing coordination.

The alternative assembly of the BAT domain makes TRPA1 more flexible in receiving reactive chemicals, as evidenced by the functional changes observed in this study. Through coexpression of the 2 isoforms containing either exon10a or exon10b BAT, the sensitivity and dynamic range of nociceptive neurons that detect reactive chemicals would be finely tuned to produce appropriate responses for the ecological niches of the organisms. In this regard, it would be interesting to examine if the 2 isoforms are present in the same neurons and what the functional outcome of the coexpression *in vivo* is. This future investigation might answer the question of why the 2 “flavors” of chemosensory functions offered by exon10a and exon10b are necessary for insect chemical nociception, while exon10b renders TRPA1 sufficiently sensitive to reactive chemicals.

## EXPERIMENTAL PROCEDURES

### Semiquantitation of Exon Segregation

It is important to prevent genomic DNA fragments from being amplified in PCR reactions to critically compare transcript levels. To this end, primers straddling 2 adjacent exons were designed and used in PCR reactions (Supplemental Fig. 1A and C). For determination of exon combinations present in *TrpA1(A)* and *TrpA1(B)* transcripts, transcript variant-specific cDNA was amplified from cDNA preparation from whole body total RNA with the use of the isoform-specific primer, either 1a-F or 1b-F, respectively, and the common reverse primer, 1st-R. The annealing site of 1st-R is positioned in exon12, which allows the amplicon to include the alternative exons, exon10a and exon10b. The first PCR product was then purified with Expin PCR purification kit (GeneAll) and subjected to the second PCR reactions primed with Nested-F and Nested-R to acquire the quantity sufficient for the DNA sequencing reaction. The nucleotide sequence of the final PCR product was determined using the Sanger method with fluorescence-linked dideoxy nucleotides (Cosmogenetech). The respective 5′ sequences of alternative exons were read and the height of signal intensities was analyzed as illustrated in Figure 1A and B. Oligonucleotides used in PCR reactions are listed in the following:

1a-F: GCCGGAACAGCAAGTATT.

1b-F: GTGGACTATCTGGAGGCG.

1st-R: GGTTCTGCGAGAGATTCATG.

Nested-F: TTCCGACAAGCATCCGTGC.

Nested-R: GTCAGCTGCTCCCATCCCAT.

### Electrophysiological Examination of Insect TRPA1 Currents in *Xenopus laevis* Oocytes

The 2-electrode voltage clamping with frog oocytes has been used as in previous studies (Du et al., 2015, Du et al., 2016a, Du et al., 2019, Du et al., 2016, Du and Kang, 2020). Briefly, surgically removed ovaries were enzymatically digested by 1.5 mg/mL collagenase for 1.5 hours at room temperature with gentle rocking. One day after injection of 50 nL of *TrpA1* cRNAs, oocytes were perfused in the recording solution (96 NaCl, 1 KCl, 1 MgCl_2_, 5 4-(2-hydroxyethyl)-1-piperazineethanesulfonic acid, pH 7.6 in mM) during current recording. Typical membrane potentials expressing TRPA1 isoforms were between −10 and −50 mV. Increasing concentrations of NMM were serially applied to the saturating activity at the respective concentration with the voltage clamped at −60 mV under the control of the GeneClamp 500B amplifier (Molecular Devices). In case the response did not saturate within 10 minutes, the next concentration of NMM was applied. Responses to NMM were normalized with respect to the current obtained with the highest concentration and fitted to the Hill equation using SigmaPlot 12 to obtain EC50s.

## Author Contributions

**Eun Jo Du:** Validation, Investigation, Formal analysis, Data curation, Conceptualization. **MinHyuk Lee:** Validation, Resources, Project administration, Data curation. **Seon Yeong Kim:** Validation, Resources, Data curation. **Se Hoon Park:** Validation, Resources, Data curation. **Hye-Jung Ohk:** Writing—review and editing, Visualization, Resources. **KyeongJin Kang:** Writing—review and editing, Writing—original draft, Supervision, Project administration, Methodology, Investigation, Funding acquisition, Formal analysis, Data curation, Conceptualization.

## Declaration of Competing Interests

The authors declare that they have no known competing financial interests or personal relationships that could have appeared to influence the work reported in this paper.

## Appendix A Supplemental material

**Supplemental Fig. 1.** Schemes of *TrpA1* gene structures and TRPA1 primary structures. (A) The gene structure of *TrpA1* in the *Drosophila* genome. (B) Scheme of TRPA1 channel protein domains. N-term, the amino-terminus; TM, the transmembrane domains; C-term, the carboxy-terminus. (C) The gene structure of *TrpA1* of *A. gambiae*. (D) Scheme of agTRPA1 protein domain organization. Boxes indicate exons. Blue and red colors were used to mark coding sequences of alternative exons. Annealing sites of primers used for RT-PCR were illustrated in relation to the gene structure. Relative location of alternative domains was indicated by white boxes.

.

**Supplemental Fig. 2.** Typical NMM dose dependences of *Drosophila* and *Anopheles* TRPA1 isoforms and human TRPA1. RR, ruthenium red (an inhibitor of TRPA1).

.

**Supplementary Fig. 3.** Sequence alignments of exon10-encoded domains deduced from insect genomes. D. mel., *Drosophila melanogaster*; D. ana., *Drosophila ananassae*; D. vir., *Drosophila virilis*; D. grim., *Drosophila grimshawi*; A. gam., *Anopheles gambiae*; A. aeg., *Aedes aegypti*; C. quin., *Culex quinquefasciatus*; M. des., *Mayetiol destructor*; R. prol., *Rhodnius prolixus*; B. mori., *Bombyx mori*.
