# Supplementary figures and images for: Linkage of alternative exon assembly in Drosophila TrpA1 transcripts
Source: Mol Cells. 2024 Sep 11;47(10):100110. doi: 10.1016/j.mocell.2024.100110 (PMC11471635; doi:10.1016/j.mocell.2024.100110)

Supp. Fig. 1

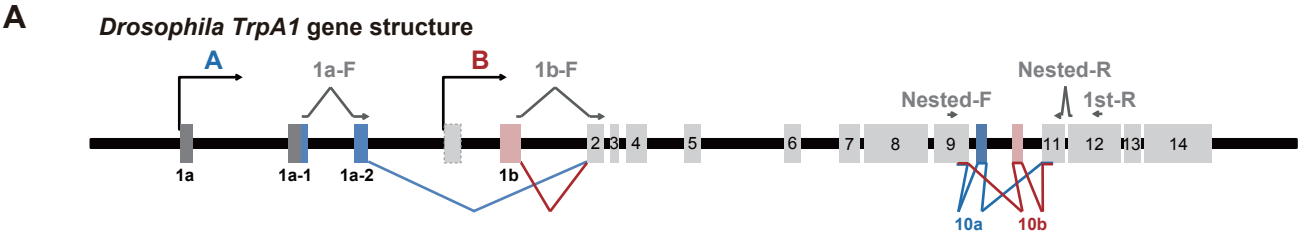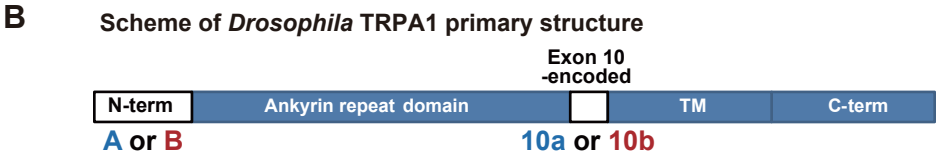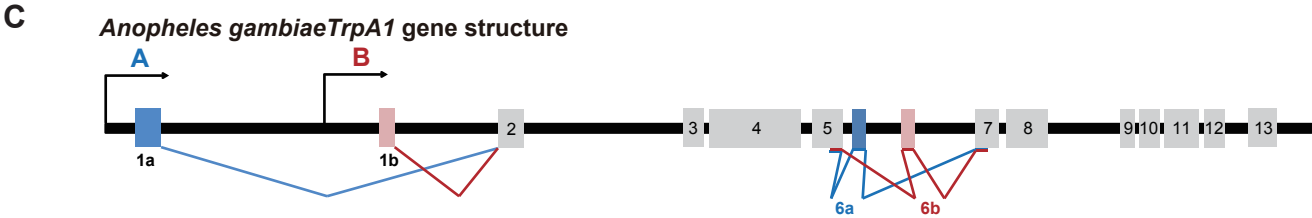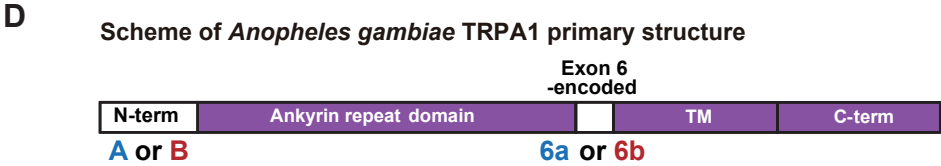

Supplement: Supplementary file 1 — Supplemental Fig. 1. Schemes of TrpA1 gene structures and TRPA1 primary structures. (A) The gene structure of TrpA1 in the Drosophila genome. (B) Scheme of TRPA1 channel protein domains. N-term, the amino-terminus; TM, the transmembrane domains; C-term, the carboxy-terminus. (C) The gene structure of TrpA1 of A. gambiae. (D) Scheme of agTRPA1 protein domain organization. Boxes indicate exons. Blue and red colors were used to mark coding sequences of alternative exons. Annealing sites of primers used for RT-PCR were illustrated in relation to the gene structure. Relative location of alternative domains was indicated by white boxes. [file mmc1.pdf]

Supp. Fig. 2

A

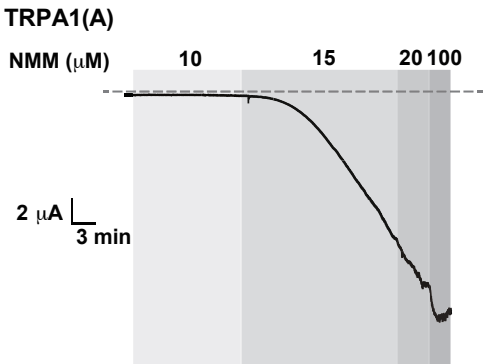

B

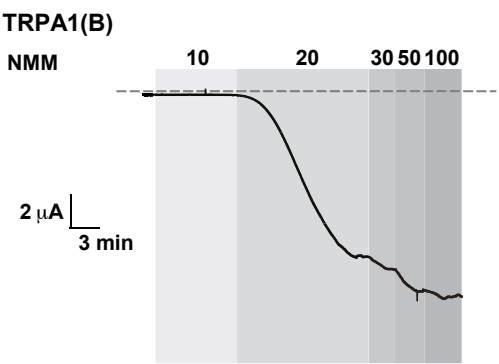

C

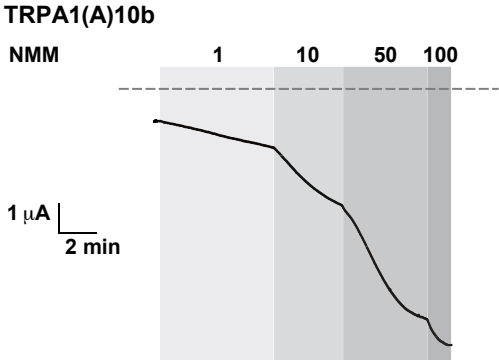

D

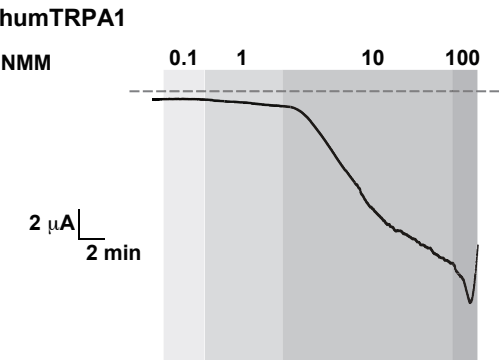

E

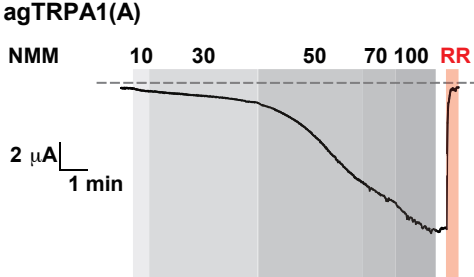

F

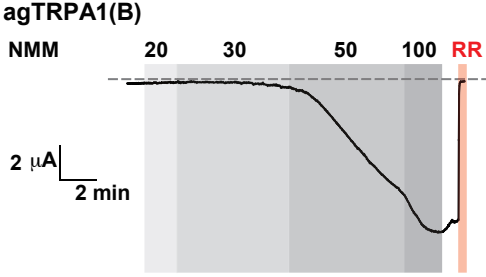

G

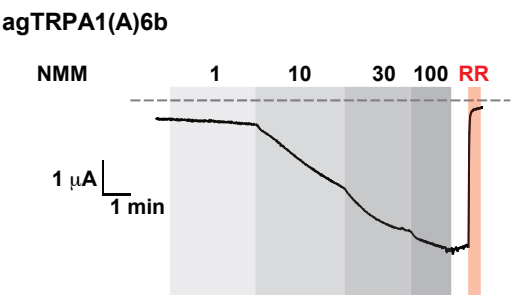

Supplement: Supplementary file 2 — Supplemental Fig. 2. Typical NMM dose dependences of Drosophila and Anopheles TRPA1 isoforms and human TRPA1. RR, ruthenium red (an inhibitor of TRPA1). [file mmc2.pdf]
