# Supplementary material for: Linkage of alternative exon assembly in Drosophila TrpA1 transcripts
Source: Mol Cells. 2024 Sep 11;47(10):100110. doi: 10.1016/j.mocell.2024.100110 (PMC11471635; doi:10.1016/j.mocell.2024.100110)
Supplement: Supplementary file 3 — Supplementary Fig. 3. Sequence alignments of exon10-encoded domains deduced from insect genomes. D. mel., Drosophila melanogaster; D. ana., Drosophila ananassae; D. vir., Drosophila virilis; D. grim., Drosophila grimshawi; A. gam., Anopheles gambiae; A. aeg., Aedes aegypti; C. quin., Culex quinquefasciatus; M. des., Mayetiol destructor; R. prol., Rhodnius prolixus; B. mori., Bombyx mori. [file mmc3.pdf]

Supp. Fig. 3

Exon10a-orthologous domain aligned

D. mel.	IKYSFAFLQC	PFMFAKIDEK	TGESITTASP	IPLPALN
D. ana.	IKYSFAFLQC	PFMFAKIDEK	TGESIATTNP	IPLPALN
D. vir.	IRYSFAFLQC	PYMFAKIDEK	TGEPIMTSNP	IPLPALN
D. grim.	IRYSFAFLQC	PYMSAKIDEK	TGEPIMTTNP	IPLPALN
A. gam.	IRYSFSCLQC	PALYAQMDAR	TGEAVQISKP	IPLPALN
A. aeg.	IKYSFSCLQC	PTMYAQMDSR	TGEAVQIFKP	IPLPALN
C. quin.	IKYSFSCLQC	PTMYAQMDSR	TGEAVQISKP	IPLPALN
M. des.	IKYSMACLQC	PTMYAQMDEK	TGEAVQISKP	IPLPALN
R. prol.	IKYNFSCLQC	TRVYSPVDQK	NGETFSQLNL	IPLPALN
B. mori	IKYSFEALCP	QLMD----ED	GTRKSQQAQQ	IPLPALN

Exon10b-orthologous domain aligned

D. mel.	IKYSFWPYQK	TPEQIEAKRK	EFNDPKWRPA	PLAVVN
D. ana.	IKYSFKFYQH	SRLEIDALRQ	ALNDPKFRPE	PLSVIN
D. vir.	IKYSFWPFQK	TPEQIEEKRK	EFNDPKWRPA	PLAVVN
D. grim.	IKYSFAPYQK	TPKQIEEKRL	EFNDAKWRPA	PLAVVN
A. gam.	IKYSFHAYQK	SQEGIAEMRK	TLNDPKWRPQ	PLHVVN
A. aeg.	IRYSFHAYQK	SQEEIDKIRK	TLNDPKWRPP	PLHVVN
C. quin.	IKYSFHAYQK	SKEEVEKIRK	ALNDPNWRQT	PLHVVN
M. des.	IKYNFNCYQY	AIEKIKKIRE	TMNDPEWRPP	PLLVIN
R. prol.	IKYSFENYQY	SPVQVSARQK	VLGNPNWRPS	PLPVVN
B. mori	IKYSFKYYQK	SKHEIEALRL	AHNDPKYRPE	PLSVIN
